# Comparative performance of humans versus GPT-4.0 and GPT-3.5 in the self-assessment program of American Academy of Ophthalmology

**DOI:** 10.1038/s41598-023-45837-2

**Published:** 2023-10-29

**Authors:** Andrea Taloni, Massimiliano Borselli, Valentina Scarsi, Costanza Rossi, Giulia Coco, Vincenzo Scorcia, Giuseppe Giannaccare

**Affiliations:** 1 Department of Ophthalmology, University Magna Graecia of Catanzaro, Catanzaro, Italy; 2 Department of Clinical Sciences and Translational Medicine, University of Rome Tor Vergata, Rome, Italy; 3 Department of Surgical Sciences, Eye Clinic, University of Cagliari, Via Università 40, 09124 Cagliari, Italy

**Keywords:** Health care, Medical research

## Abstract

To compare the performance of humans, GPT-4.0 and GPT-3.5 in answering multiple-choice questions from the American Academy of Ophthalmology (AAO) Basic and Clinical Science Course (BCSC) self-assessment program, available at https://www.aao.org/education/self-assessments. In June 2023, text-based multiple-choice questions were submitted to GPT-4.0 and GPT-3.5. The AAO provides the percentage of humans who selected the correct answer, which was analyzed for comparison. All questions were classified by 10 subspecialties and 3 practice areas (diagnostics/clinics, medical treatment, surgery). Out of 1023 questions, GPT-4.0 achieved the best score (82.4%), followed by humans (75.7%) and GPT-3.5 (65.9%), with significant difference in accuracy rates (always P < 0.0001). Both GPT-4.0 and GPT-3.5 showed the worst results in surgery-related questions (74.6% and 57.0% respectively). For difficult questions (answered incorrectly by > 50% of humans), both GPT models favorably compared to humans, without reaching significancy. The word count for answers provided by GPT-4.0 was significantly lower than those produced by GPT-3.5 (160 ± 56 and 206 ± 77 respectively, P < 0.0001); however, incorrect responses were longer (P < 0.02). GPT-4.0 represented a substantial improvement over GPT-3.5, achieving better performance than humans in an AAO BCSC self-assessment test. However, ChatGPT is still limited by inconsistency across different practice areas, especially when it comes to surgery.

## Introduction

Generative Pretrained Transformer (GPT) is a large language model (LLM) powered by artificial intelligence (AI) and developed by OpenAI. In order to understand and “generate” human-like language, LLMs are “pretrained” on a substantial volume of text data from the internet, employing a neural network architecture called “Transformer”. The latest versions of the model are GPT-3.5 and GPT-4.0, launched in March 2022 and March 2023 respectively. Both models are available and optimized for natural conversation in the ChatGPT web app; however, GPT-4.0 is currently accessed under a paid monthly subscription^1^.

The growing interest around ChatGPT has led to a considerable number of recent publications investigating the impact of AI language models in the medical environment, from both an educational and a workflow perspective^2^. In the ophthalmology setting, ChatGPT has been tested for triaging patient symptoms^3^ and for answering patient and care giver questions about specific ocular diseases^4,5^. To our knowledge, four papers analyzed the performance of LLMs in answering multiple-choice questions from simulations of board certification exams^6–11^, but only one included direct comparison between humans, GPT-3.5 and GPT-4.0 in a limited sample of American Academy of Ophthalmology (AAO) Basic and Clinical Science Course (BCSC) questions^9^.

The purpose of this study was to compare the overall performance of humans, GPT-4.0 and GPT-3.5 on a larger sample of multiple-choice questions of the AAO BCSC self-assessment program available at https://www.aao.org/education/self-assessments to all the subscribed members; furthermore, the comparative performance of these three respondents according to different categorization of the questions, including subspecialties and practice areas, was calculated.

## Methods

In this comparative cross-sectional study, the multiple-choice questions included in the self-assessment test from the clinical education section of the AAO official website (https://www.aao.org/education/self-assessments) were submitted in June 2023 to ChatGPT, using both GPT-4.0 and GPT-3.5 language models.

The multiple-choice questions are divided in 10 sets, based on subspecialty: (1) Cataract/Anterior Segment, (2) Cornea/External Disease, (3) Glaucoma, (4) Neuro-Ophthalmology/Orbit, (5) Ocular Pathology/Oncology, (6) Oculoplastics/Orbit, (7) Pediatric Ophth/Strabismus, (8) Refractive Mgmt/Intervention, (9) Retina/Vitreous, (10) Uveitis. Additionally, a masked investigator further classified questions according to practice area: (1) Diagnostics/Clinics, (2) Medical treatment, (3) Surgery. Since ChatGPT cannot analyze multimedia content, questions containing images were excluded from the study. Each consecutive multiple-choice question was entered in separate chat-rooms. In almost every response, ChatGPT discussed the topic of the submitted question, and then selected an option. If ChatGPT did not choose any of the provided answers, investigators resubmitted the question in a new chat, adding the prompt “You must choose one” after the question mark, to force a definite choice.

Upon submitting the multiple-choice option selected by ChatGPT in the self-assessment test, the correct answer is revealed. Results for humans were obtained from past completed examinations stored in the AAO website, rather than through direct engagement of human participants: after answering each question, the percentage of humans who chose each distinct option are revealed on the website. Regrettably, the AAO does not provide any information about the number of human respondents, their level of education or degree of proficiency in ophthalmology. Questions answered incorrectly by more than 50% of human users were categorized as difficult, while those answered correctly by at least 95% of humans were considered easy.

AAO questions, ChatGPT outputs and chosen answers, along with the percentage of humans who selected each multiple-choice option, were recorded. As secondary outcome, the word counts for questions and for responses by ChatGPT were calculated to assess (1) AI verbosity, (2) word count response differences for correct or incorrect answers and (3) correlation between the length of the questions and the length of the discussion provided by the AI.

All data were entered into an electronic database via Microsoft Office Excel 365 (Microsoft Corp., Redmond, WA) and analyzed with IBM SPSS Statistics (version 29.0; IBM, Armonk, New York). Ethical approval and informed consent were not required, since the study did not involve human participants.

### Statistical analysis

The number of correct answers from humans, GPT-4.0 and GPT-3.5 was compared by Chi-square (${\upchi }^{2}$) and Fisher's exact test for the analysis of categoric variables. This analysis was repeated for all subspecialty sets and practice areas. The Kolmogorov–Smirnov test was performed to determine the normality of data. Paired Student t test for normally distributed variables was used to compare word counts between the responses provided by GPT 4.0 and GPT 3.5. Independent Student t test was performed to compare word counts between the responses provided by ChatGPT for questions answered correctly and those answered wrong. Univariate analysis was used to compare the word count of questions and answers from ChatGPT. When appropriate, values were expressed as mean ± standard deviation. Word count for ChatGPT responses was always rounded down. All tests were two-sided and a *P* value < 0.05 was considered statistically significant.

### Ethics approval

In this observational study no humans were recruited. The local ethical committee (Comitato Etico Area Centro, Regione Calabria) exempted this study from the requirement for ethical approval.

## Results

As of June 2023, the self-assessment test at https://www.aao.org/education/self-assessments included 1073 multiple-choice questions; 50 (4.7%) were excluded from the analysis because they incorporated images. In total, 1023 questions were submitted to both GPT-4.0 and GPT-3.5. Table 1 presents a detailed summary of the questions distribution across different subspecialities and practice areas.

**Table 1:** Questions distribution across the different subspecialties and practice areas.

Subspecialty	Practice area			
	Diagnostics/clinics	Medical treatment	Surgery	All
	Number (%)	Number (%)	Number (%)	Number (%)
Cataract/anterior segment	43 (4.2%)	10 (1.0%)	63 (6.2%)	116 (11.3%)
Cornea/external disease	67 (6.5%)	27 (2.6%)	4 (0.4%)	98 (9.6%)
Glaucoma	71 (6.9%)	57 (5.6%)	8 (0.8%)	136 (13.3%)
Neuro-ophthalmology/orbit	76 (7.4%)	6 (0.6%)	0 (0.0%)	82 (8.0%)
Ocular pathology/oncology	34 (3.3%)	0 (0.0%)	1 (0.1%)	35 (3.4%)
Oculoplastics/orbit	63 (6.2%)	28 (2.7%)	25 (2.4%)	116 (11.3%)
Pediatric ophth/strabismus	75 (7.3%)	40 (3.9%)	7 (0.7%)	122 (11.9%)
Refractive Mgmt/intervention	36 (3.5%)	13 (1.3%)	19 (1.9%)	68 (6.6%)
Retina/vitreous	98 (9.6%)	20 (2.0%)	10 (1.0%)	128 (12.5%)
Uveitis	83 (8.1%)	34 (3.3%)	5 (0.5%)	122 (11.9%)
All	646 (63.1%)	235 (23.0%)	142 (13.9%)	1023 (100.0%)

To ensure that our study was adequately powered to detect meaningful differences between groups of responders, we conducted a power analysis based on the percentage of questions answered correctly by GPT-4.0 (84.3%), GPT-3.5 (69.5%) and humans (72.9%) in a previous study by Lin et al., who also employed the multiple-choice questions of the AAO BCSC program^9^. The analysis indicated that to achieve 0.80 power at a 0.05 significance level, the study should include a sample size of 252 questions for comparisons between GPT-4.0 and GPT-3.5, 404 questions for GPT-4.0 vs humans, and a notably larger sample of 5566 questions for GPT-3.5 vs humans.

In some cases, ChatGPT did not select an answer on the first query (28 questions [2.73%] for GPT-4.0 and 37 [3.61%] for GPT-3.5; ${\upchi }^{2}$ = 1.29, *P* = 0.25). Upon resubmitting the questions with the additional prompt “You must choose one”, a multiple-choice option was always selected.

Overall, GPT-4.0 and GPT-3.5 answered correctly to 843 (82.4%) and 674 (65.9%) questions respectively, while the mean percentage of questions answered correctly by humans was 75.7 ± 17.2%, corresponding to 774 ± 176 (95% CI, 763–785) questions. The number of correct answers by GPT-4.0 was significantly higher than both human users (${\upchi }^{2}$ = 14.02, *P* = 0.0002) and GPT-3.5 (${\upchi }^{2}$= 72.82, *P* < 0.0001). Conversely, GPT-3.5 compared unfavorably to humans (${\upchi }^{2}$ = 23.65, *P* < 0.0001).

### Subspecialty groups

GPT-3.5 performance was significantly variable across the 10 different subspecialties (${\upchi }_{9}^{2}$ = 31.64, *P* = 0.0002). In particular, GPT-3.5 obtained the highest percentage of correct answers in Ocular Pathology/Oncology (27 out of 35, 77.1%) and the lowest in Pediatric Ophthalmology/Strabismus (65 out of 122, 53.3%). Conversely, GPT-4.0 and humans showed more consistent results, with no significant difference across subspecialty groups (${\upchi }_{9}^{2}$ = 13.24, *P* = 0.15 and ${\upchi }_{9}^{2}$ = 3.06, *P* = 0.96 respectively).

Despite the fact that GPT-4.0 performed better than humans in all subspecialties, the difference was statistically significant only in Glaucoma (${\upchi }^{2}$ = 5.00, *P* = 0.02) and Oculoplastics/Orbit (${\upchi }^{2}$ = 4.04, *P* = 0.04). A post-hoc power analysis was performed to check whether the lack of significant differences in other subspecialties could be determined by an insufficient sample size. The power analysis confirmed this suspicion, showing low statistical power for the comparisons in most subspecialty groups.

**Figure 1:**
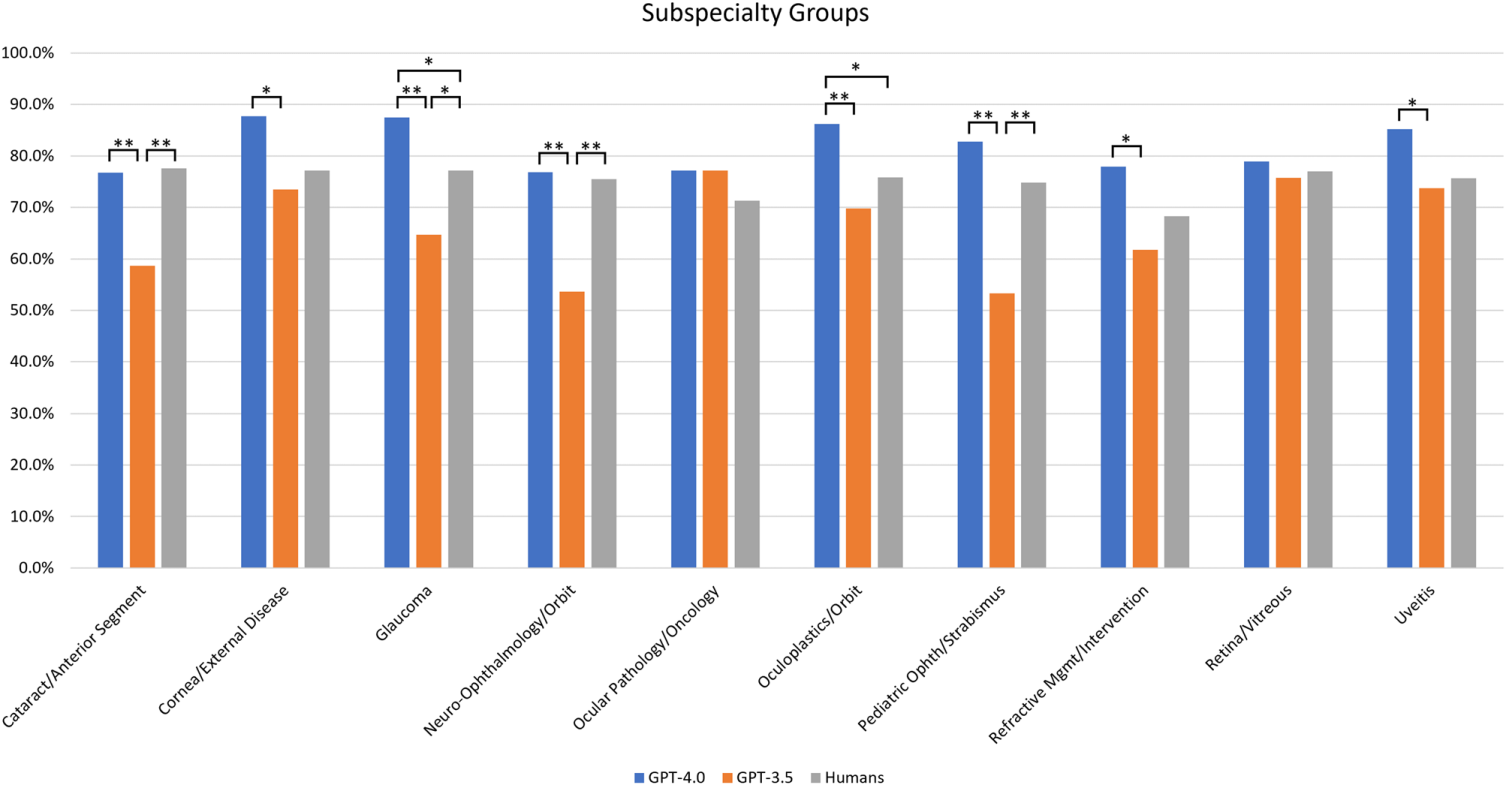
Percentage of correct answers for GPT-4.0, GPT-3.5 and humans according to subspecialty groups. (*) indicates a *P* value < 0.05; (**) indicates a *P* value < 0.01.

GPT-3.5 showed worse performance, obtaining significantly lower scores than humans in Cataract/Anterior Segment (${\upchi }^{2}$ = 9.64, *P* = 0.002), Glaucoma (${\upchi }^{2}$ = 5.11, *P* = 0.02), Neuro-Ophthalmology/Orbit (${\upchi }^{2}$ = 8.56, *P* = 0.003) and Pediatric Ophthalmology/Strabismus (${\upchi }^{2}$ = 12.29, *P* = 0.0004). Table 2 and Fig. 1 present results for humans, GPT-4.0 and GPT-3.5 across all subspecialties.

**Table 2:** Number and percentage of correct answers for humans, GPT-4.0 and GPT-3.5 across different subspecialties and practice areas.

Question classification	Questions number (%)	Humans mean ± SD (mean ± SD%)	GPT-4.0 correct answers (%)	GPT-3.5 correct answers (%)	GPT-4.0 vs. humans P value (post-hoc power)	GPT-3.5 vs. Humans P value (post-hoc power)	GPT-4.0 vs. GPT-3.5 P value (post-hoc power)
All	1023 (100.0%)	774 ± 175 (75.7 ± 17.1%)	843 (82.4%)	674 (65.9%)	0.0002* (0.96)	< 0.0001* (1.00)	< 0.0001* (1.00)
Subspecialty							
Cataract/anterior segment	116 (11.3%)	90 ± 18 (77.6 ± 16.3%)	89 (76.7%)	68 (58.6%)	0.8757 (0.03)	0.0019* **(0.88)**	0.0032* **(0.84)**
Cornea/external disease	98 (9.6%)	75 ± 15 (77.1 ± 16.1%)	86 (87.8%)	72 (73.5%)	0.0508 (0.50)	0.5510 (0.08)	0.0114* (0.72)
Glaucoma	136 (13.3%)	104 ± 22 (77.1 ± 16.7%)	119 (87.5%)	88 (64.7%)	0.0253* (0.61)	0.0237* (0.61)	< 0.0001* **(1.00)**
Neuro-ophthalmology/orbit	82 (8.0%)	61 ± 12 (75.5 ± 15%)	63 (76.8%)	44 (53.7%)	0.8431 (0.04)	0.0034* **(0.84)**	0.0018* **(0.88)**
Ocular pathology/oncology	35 (3.4%)	24 ± 6 (71.3 ± 18.6%)	27 (77.1%)	27 (77.1%)	0.5754 (0.08)	0.5754 (0.08)	1.000 (N/A)
Oculoplastics/orbit	116 (11.3%)	88 ± 19 (75.9 ± 17.1%)	100 (86.2%)	81 (69.8%)	0.0444* (0.52)	0.3015 (0.18)	0.0026* **(0.86)**
Pediatric ophth/strabismus	122 (11.9%)	91 ± 20 (74.8 ± 16.7%)	101 (82.8%)	65 (53.3%)	0.1279 (0.33)	0.0004* **(0.94)**	< 0.0001* **(0.99)**
Refractive Mgmt/intervention	68 (6.6%)	46 ± 13 (68.3 ± 19.8%)	53 (77.9%)	42 (61.8%)	0.2052 (0.24)	0.4236 (0.12)	0.0398* (0.53)
Retina/vitreous	128 (12.5%)	98 ± 22 (77 ± 17.9%)	101 (78.9%)	97 (75.8%)	0.7141 (0.05)	0.8173 (0.04)	0.5504 (0.09)
Uveitis	122 (11.9%)	92 ± 21 (75.7 ± 17.5%)	104 (85.2%)	90 (73.8%)	0.0591 (0.46)	0.7337 (0.05)	0.0264* (0.60)
Practice area							
Diagnostics/clinics	646 (63.1%)	487 ± 111 (75.4 ± 17.2%)	541 (83.7%)	440 (68.1%)	0.0002* **(0.96)**	0.0035* **(0.83)**	< 0.0001* **(1.00)**
Medical treatment	235 (23.0%)	181 ± 40 (76.9 ± 16.9%)	196 (83.4%)	153 (65.1%)	0.079 (0.42)	0.0047* **(0.81)**	< 0.0001* **(1.00)**
Surgery	142 (13.9%)	106 ± 24 (74.7 ± 17.2%)	106 (74.6%)	81 (57.0%)	0.9989 (0.03)	0.0017* **(0.89)**	0.0017* **(0.88)**

*A P value < 0.05 was considered statistically significant. Post-hoc power > 0.80 has been highlighted in bold.

### Practice area groups

**Figure 2:**
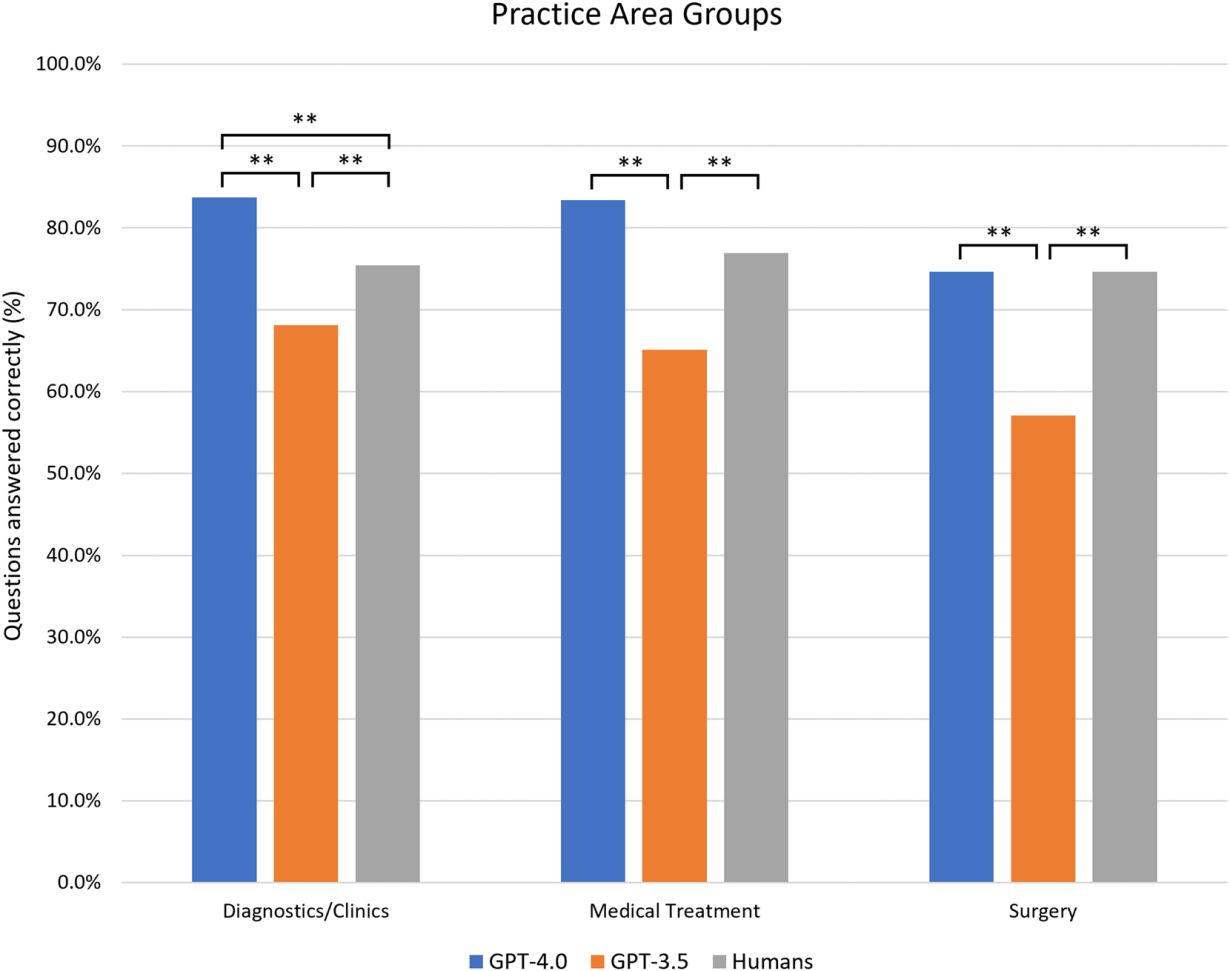
Percentage of correct answers for GPT-4.0, GPT-3.5 and humans according to practice area groups. (**) indicates a *P* value < 0.01.

GPT-4.0 and GPT-3.5 showed significant difference in performance among practice areas (${\upchi }_{2}^{2}$ = 6.86, *P* = 0.03 and ${\upchi }_{2}^{2}$ = 6.43, *P* = 0.04 respectively). GPT-4.0 and GPT-3.5 obtained the best scores in Diagnostics/Clinics (541 and 440 out of 646, 83.7% and 68.1% respectively) and the worst in Surgery (106 and 81 out of 142, 74.6% and 57.0% respectively). Conversely, human users achieved better consistency, with no significant difference across practice areas (${\upchi }_{2}^{2}$ = 0.31, *P* = 0.86). More specifically, GPT-4.0 performed better than humans in Diagnostics/Clinics (${\upchi }^{2}$ = 13.78, *P* = 0.0002) and Medical treatment (${\upchi }^{2}$ = 3.08, *P* = 0.08), while GPT-3.5 obtained significantly lower scores than humans in all practice areas (always *P* < 0.01). Table 2 and Fig. 2 present results across all practice areas.

### Difficulty groups

In total, 92 out of 1023 (9.0%) questions were answered incorrectly by more than 50% of human users. The majority of these questions were included in the Refractive (16 out of 92, 23.5%) and the Ocular Pathology/Oncology (6 out of 35, 17.1%) subspecialties. In these difficult cases, the mean percentage of correct answers for humans was 40.1 ± 7.5%. Both GPT-4.0 and GPT-3.5 performed better than humans in these questions (49 out of 92, 53.3%; 44 out of 92, 47.8% respectively), but without reaching significancy (always *P* > 0.05). Difference between GPT-4.0 and GPT-3.5 was also not significant (${\upchi }^{2}$ = 0.54, *P* = 0.46).

**Figure 3:**
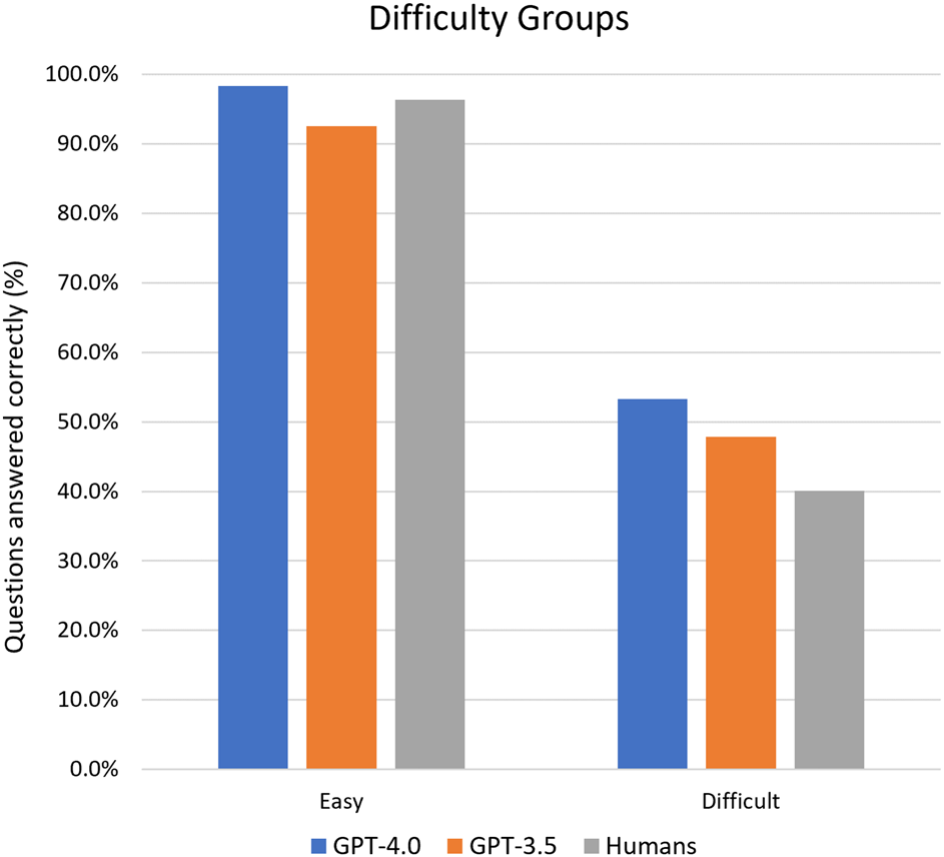
Percentage of correct answers for GPT-4.0, GPT-3.5 and humans for easy and difficult questions.

Overall, 120 out of 1023 (11.7%) questions were answered correctly by 95% or more humans. To evaluate major mistakes, the number of times ChatGPT chose the wrong answer to these questions was tracked. The mean percentage of correct answers for humans in this subset was 96.4 ± 1.2%; only 2 questions were answered incorrectly by GPT-4.0 (98.3%), and 9 by GPT-3.5 (92.5%). There was no significant difference between both GPT models and humans (*P* > 0.2), however the difference between GPT-4.0 and GPT-3.5 almost reached a significant level (*P* = 0.059). Figure 3 presents results for easy and difficult questions.

### Word count analysis

On average, the word count for answers provided by GPT-4.0 was significantly lower than those produced by GPT-3.5 (160 ± 56 [95% CI, 156–163] and 206 ± 77 [95% CI, 201–211] respectively, *P* < 0.0001). Furthermore, there was a significant increase in word count in responses generated for questions that were answered incorrectly compared to those that were answered correctly, for both GPT-4.0 (156 ± 54 vs 176 ± 61, *P* < 0.0001) and GPT-3.5 (202 ± 73 vs 213 ± 78, *P* = 0.02). According to univariate analyses, the word count for responses of both GPT-4.0 and GPT-3.5 was positively correlated to the word count of the questions (always *P* < 0.001, β = 0.212 and β = 0.153 respectively).

## Discussion

This study compared the performance of humans, GPT-4.0 and GPT-3.5 in a large ophthalmology test based on multiple-choice questions. Instead of running a simulation inside the AAO BCSC web app, which includes 260 random questions extracted from a database of 4500 + entries, the more comprehensive self-assessment test available at https://www.aao.org/education/self-assessments, containing a total of 1073 questions, has been evaluated. These questions, extracted from the same large database of the BCSC program, are always the same for all users, thus improving the reproducibility of these results when compared to future studies. To our knowledge, this study included the largest number of ophthalmological questions submitted to ChatGPT (Pubmed, Keywords: GPT + Ophthalmology; Years: 2021–2023).

The difference in correct answers among the three study groups was statistically significant: GPT-4.0 achieved the best score (82.4%), followed by humans (75.7%) and GPT-3.5 (65.9%). Interestingly, both GPT-4.0 and GPT-3.5 showed less consistent results across different practice areas compared to humans. Diagnostics/Clinics was the easiest practice area (83.7% and 68.1% respectively), with Medical treatment following closely (83.4% and 65.1%). Instead, Surgery was the hardest area for both models (74.6% and 57.0%). It is possible to hypothesize that answers for clinical questions are easier to deduct from web resources, while surgery may involve highly specialized techniques and protocols that may not be well-represented in the data AI language models were trained on. Likewise, surgical knowledge often relies on images or videos, which were not included in the training database. Concerning subspecialties, GPT-3.5 also demonstrated significant inconsistencies, whereas GPT-4.0 achieved a degree of consistency much closer to human performance.

The performance of GPT-4.0 and GPT-3.5 was further analyzed by considering only difficult questions, defined as questions answered incorrectly by more than 50% of human users. Remarkably, both models performed better than humans in this challenging subset, but the difference was not statistically significant (40.1% for humans, 53.5% for GPT-4.0 and 47.8% for GPT-3.5). The outcomes for the easiest questions, defined as those answered correctly by 95% or more humans, was comparable among AI models and human users (96.4% for humans, 98.3% for GPT-4.0 and 92.5% for GPT-3.5).

Although in multiple-choice questions the word count of ChatGPT responses has no impact on the selection of a correct answer, succinctness still plays a significant role in user experience, especially in the healthcare environment. Clear and concise answers are generally preferred, as long as the complexity of the topic is respected. Overall, the length of ChatGPT answers was directly proportional to word count of the questions, with GPT-4.0 outputs being significantly more succinct than GPT-3.5 (160 ± 56 vs. 206 ± 77 words). On the other hand, responses were significantly longer for questions answered incorrectly in both language models. Although explaining the technical reasons behind this behavior goes beyond the scope of this study, it was observed that excessive verbosity was often associated with evasive and generic responses, hinting at potential uncertainties in the resolution of the questions.

Other authors have performed similar research to assess the potential of ChatGPT in ophthalmology. Lin et al. compared the performance of GPT-4.0, GPT-3.5 and humans in the setting of the AAO BCSC Self-Assessment Program Simulated Exam (260 questions). The three groups of respondents scored 84.3%, 69.5% and 72.9% respectively. These results are consistent with our findings (+ 1.9%, + 3.6% and − 2.8% respectively); however, Lin et al. marked non-answers as incorrect, while we included the additional prompt “You must choose one” to force an answer. Such difference in design limits the relatability of the comparison between the two studies. Furthermore, Lin et al. took an alternative approach to assess the difficulty of each question: instead of evaluating difficulty based on the percentage of human users who answered correctly to the questions, a masked investigator classified questions as first-order (fact recall) or higher-order (evaluative/analytical tasks) problem-solving^9^.

More recently, Moshirfar et al. evaluated the performance of GPT-4.0, GPT-3.5 and humans in answering 467 questions from the StatPearls question bank, respectively scoring 73%, 55% and 58%. While a direct comparison is not completely appropriate, since two different datasets of questions were used, scores for all respondents were lower than what we found (− 9.4%, − 10.9% and − 17.7% respectively). In this study, the difficulty score, ranging from 1 to 4, was provided by StatPearls. Results for humans were not affected by difficulty grading, while GPT-3.5 showed a notable decline in performance as the level of difficulty increased. This trend was much less evident for GPT-4.0^12^. A limitation of this study lies in the fact that the AAO self-assessment test exclusively reveals the percentage of humans who selected a certain multiple-choice option, without disclosing the number of respondents and their degree of knowledge. Additionally, the reproducibility of ChatGPT answers was not assessed herein, although a previous study by Antaki et al. reported almost perfect repeatability^6^.

Despite the great potential of generative AI, many challenges and outstanding issues are emerging. First, the source of the massive amount of data used to train LLMs was not subjected to rigorous quality control, with high risk of false or misleading information. In this regard, it is crucial to understand that ChatGPT does not really “understand” questions; instead, it answers based on probability correlations of text found within the training datasets and statistical patterns^13^. Low quality data may introduce algorithmic bias, possibly reinforcing old, outdated knowledge. Finally, AI can generate hallucinations, consisting of completely fabricated information, factual inaccuracies, logical inconsistencies or nonsensical responses^13,14^. Malevolent uses of ChatGPT are also conceivable, posing a serious threat for the integrity of scientific research. For instance, many authors reported how ChatGPT could produce seemingly authentic scientific manuscripts or abstracts, avoiding plagiarism checks^15–18^.

In conclusion, GPT-4.0 represented a substantial improvement over GPT-3.5, achieving higher performance compared to humans in the self-assessment tests for the American Board of Ophthalmology examination. However, ChatGPT is still limited by performance inconsistency across different practice areas, especially when it comes to surgical/practical knowledge. Currently, the utility of ChatGPT extends beyond that of a comprehensive repository of ophthalmological knowledge. AI based language models demonstrated remarkable capabilities for real-time data interpretation, with the potential to revolutionize teleophthalmology services by providing almost instant responses to patient queries^4,5^, as well as assisting ophthalmologists in clinical decision-making, research and administration^19,20^. Acknowledging ChatGPT shortcomings, such as hallucinations, and potential abuses, will be crucial for the successful integration of this technology in the medical setting^16,21^.

## Data Availability

Data is available upon request by contacting Andrea Taloni, MD (email: taloni.oculistica@gmail.com).
